# Exercise Predicts a Good Night’s Sleep: Preliminary Findings from a UCLA Study of First-Episode Schizophrenia

**DOI:** 10.3390/bs13020088

**Published:** 2023-01-21

**Authors:** Kenneth L. Subotnik, Sarah C. McEwen, Joseph Ventura, Luana Rene Turner, Yurika Sturdevant, Trudy L. Niess, Laurie R. Casaus, Margaret G. Distler, Michael F. Zito, Gerhard S. Hellemann, Clara D. Nguyen, Keith H. Nuechterlein

**Affiliations:** 1 Department of Psychiatry and Biobehavioral Sciences, UCLA, Los Angeles, CA 90095, USA; 2 atai Life Sciences, San Diego, CA 92130, USA; 3 Department of Psychology, UCLA, Los Angeles, CA 90095, USA

**Keywords:** schizophrenia, sleep quality, aerobic exercise

## Abstract

Background: Physical exercise can improve sleep quality in the general population. Understanding the negative impact of poor sleep quality on multiple domains of functioning among persons with schizophrenia is a new frontier of exploration. It is also imperative to investigate non-pharmacologic methods to improve sleep quality as these approaches may not carry the side effect burdens associated with medication. Objective: We examined the relationship between regular physical exercise and sleep quality among participants in an intervention consisting of both cognitive training and exercise. Methods: Participants (N = 48) were schizophrenia patients who had a first psychotic episode within two years of study entry. Participants received 4 h/week of internet-based cognitive training and an aerobic exercise program over a 6-month period. Sleep was assessed with the Pittsburgh Sleep Quality Index at baseline and six months later. Results: During the 3 months prior to the 6-month follow-up sleep assessment, participants completed an average of 12.6 group exercise sessions and an average of 12.9 individual at-home exercise sessions. A significant relationship between the number of exercise sessions and global sleep quality was seen at month six, r = −0.44, df = 39, *p* < 0.01. Group exercise frequency was also associated with improvement in global sleep quality over the six-month intervention, t(34) = −2.84, *p* = 0.008. Conclusion: We demonstrated that a group of young adults with schizophrenia can be engaged in a regular exercise program, even during the tumultuous early course of the disorder. The number of exercise sessions in which they participated was associated with better sleep quality at six months and pre–postintervention improvement in sleep quality. Key message: Improved sleep quality appears to be a benefit of regular exercise among individuals with serious mental illness.

## 1. Background

Sleep disturbance is common among individuals with mental health disorders [1]. Further, sleep difficulties in people with schizophrenia can lead to daytime fatigue and a sedentary lifestyle, leading to greater physical health morbidity [2]. Poor sleep quality can lead to cognitive impairment leading to functional impairment [3]. Additionally, poor sleep quality among individuals with serious mental disorders [4] might exacerbate health burdens caused, in part, by antipsychotic medications. Many antipsychotic medications can contribute to symptoms of metabolic syndrome, such as weight gain, insulin resistance, high blood pressure, and an adverse lipid profile. Inadequate or poor sleep quality can lead to and exacerbate these same metabolic syndrome outcomes. Higher rates of these medical problems, combined with poorer medical monitoring and care, may all contribute to a premature mortality of up to 15 years for people with severe mental illness [5].

Poor sleep quality, with its physical and economic toll, is recognized as a major public health issue. The National Institutes of Health Sleep Research Plan (NIHSRP) has established strategic goals to strengthen the understanding of sleep disorders and to develop strategies to alleviate sleep problems [6]. Among the goals of the NIHSRP is to investigate novel therapeutic approaches to improve sleep quality, such as complementary and integrative health approaches, which include physical exercise.

Although aerobic exercise interventions are underutilized in standard clinical care of seriously mentally ill patients, research suggests the promise of this treatment modality. A meta-analysis of 22 studies examining exercise interventions on health and symptom outcomes found a significant beneficial impact on several health metrics among individuals living with schizophrenia [7]. Further, our previously completed pilot study [8] and prior RCT [9] demonstrated that adding aerobic exercise to cognitive training improves both neurocognition and everyday functioning in our first-episode schizophrenia samples. We believe that exercise has promising effects that go beyond cognition and functional outcome. An ongoing confirmatory RCT has allowed an opportunity to examine the impact of regular exercise on another factor crucial to quality of life, namely, sleep quality. We hypothesized that the frequency of exercise sessions would be associated with sleep quality.

## 2. Methods

### 2.1. Participants

Study participants were enrolled in the UCLA Aftercare Research Program from December 2016 through March 2022. This research was approved by the UCLA IRB and all participants provided written informed consent. Participants were recruited from a variety of Los Angeles area psychiatric hospitals and clinics, as well as through clinician referrals. Study inclusion criteria for entry into the Aftercare Research Program were: (1) a recent onset of psychotic illness, with the start of the first major psychotic episode within the last 2 years; (2) a DSM-5 diagnosis of schizophrenia, schizoaffective disorder (depressed type), or schizophreniform disorder; (3) 18 to 45 years of age; (4) sufficient acculturation and fluency in the English language to avoid invalidating research measures; and (5) a residence within commuting distance of the UCLA Aftercare Research Program. Study exclusion criteria were: (1) a known neurological disorder or significant CNS traumatic injury; (2) moderate or severe alcohol or substance use disorder within the six months prior to the first episode or evidence that substance abuse makes the schizophrenia diagnosis ambiguous; and (3) premorbid IQ below 70. 

After entry into the UCLA Aftercare Research Program, participants were stabilized on antipsychotic medication sufficiently for participation in the group interventions, and then randomly assigned to either the Cognitive Training Plus Exercise (CT&E) condition or the Cognitive Training Plus Healthy Living Group (CT&HLG) condition. Because this report focused on the amount of aerobic exercise completed among participants in the CT&E group, only the data from the participants in that intervention group are presented here. Baseline assessments were completed just prior to randomization, typically around three months after study entry. The aerobic exercise and cognitive training interventions lasted 6 months.

### 2.2. Interventions

All patients were prescribed second-generation antipsychotic medication, provided regular visits with the treating psychiatrist, offered individual case management, and participated in the exercise intervention described below. Details on the full RCT can be found at ClinicalTrials.gov, Identifier: NCT02823041. The interventions for the CT&E condition, including the computerized cognitive training (CT) provided to all participants, were the same as in the pilot RCT precursor to the present study and are described in greater detail in Nuechterlein et al. (2022) [10].

### 2.3. Exercise Program

In addition to the CT sessions, the patients also participated in a 24-week progressive aerobic conditioning and strengthening exercise program that was designed for the pilot RCT [10]. The CT&E exercise program was designed to meet the ACSM and AHA adult recommendations of at least 150 min of moderate aerobic exercise with at least 2 days of muscle conditioning exercises per week. The protocol required two 45 min group exercise sessions/week and two 30 min sessions/week of at-home exercise. A certified exercise instructor led the group exercise. The exercise sessions included 39 min of combined moderate-intensity aerobic conditioning (1 min intervals) and moderate-to-high-intensity strength and callisthenic conditioning (1 min intervals), followed by a cardio burst (3 min interval). The aerobic conditioning was preceded by a dynamic warm-up (7.5 min) and followed by a cool-down period (7.5 min). Participants were instructed on proper form and technique for five different exercises at the start of each session and completed three rounds of five sets of the aerobic and strength training intervals. Aerobic exercise intensity was monitored via a wireless activity and heart rate monitor (Scosche Rhythm+) to ensure patients were exercising in their individualized target heart rate zone. The exercise program was individually tailored to the patient’s current physical abilities with intensity incrementally increased over time. To encourage adherence to the group and individual (at-home) exercise programs, we employed a number of methods to motivate participation including automated individual text message reminders, an enthusiastic and experienced exercise trainer, individualized homework plans, monetary rewards ($2 for each group session completed, $5 for each individual session), social incentives, goal setting during weekly group sessions, and meetings with patients and their family members to provide support at home and to increase at-home exercise session adherence. 

### 2.4. Measures

#### 2.4.1. Pittsburgh Sleep Quality Index (PSQI)

The Pittsburgh Sleep Quality Index [11] (PSQI) is a self-report scale of sleep quality administered in an interview format. Respondents are asked to consider the past month in their report. The PSQI yields seven algorithm-derived subscales and a total score. Lower scores indicate better sleep quality. The Overall Sleep Quality score can range from 0 to 21, with more than 5 considered to be poor sleep quality. The PSQI was completed at baseline and at the end of the 6-month randomized treatment. For our analyses, we used the PSQI ratings that covered the 1-month period prior to the 6-month point.

#### 2.4.2. Number of Exercise Sessions Completed

In order to examine the effects of exercise that was were proximal to the 6-month-point PSQI ratings, we tallied the number of exercise sessions during months 4 through 6. We counted separately the number of sessions completed at the clinic in exercise groups and the number of exercise sessions completed individually at home. We allowed excused absences if a patient was physically ill and unable to exercise during a given period.

## 3. Results

The impact of participation in the aerobic exercise groups and exercise homework on sleep quality was examined for participants in the exercise condition (CT&E). These 39 participants had a mean age of 23.7 (SD = 6.4) years, a mean of 12.7 (SD = 0.6) years of education, were predominantly male (65.5%), and had diverse racial (White, 27.6%; Asian, 21.5%; Black/African American, 11.5%; Pacific Islander/Hawaiian, 3.4%; two or more races, 36.0%) and ethnic (Hispanic, 32.6%) backgrounds. Their first psychotic episode began an average of 8.9 months prior to project entry. The participants had an age, educational level, and sex distribution typical of individuals with a first episode of psychosis, and a racial and ethnic makeup that was representative of the Greater Los Angeles area.

Participants in the exercise group completed an average of 12.6 (SD = 6.3) group exercise sessions during months four through six of the study, and 12.9 (SD = 13.3) individual exercise homework sessions. Consistent with our hypothesis, a significant relationship was observed between the number of group exercise sessions and the global sleep quality score at month six, r = −0.44, N = 39, *p* = 0.01. Among the PSQI subscales, the number of group exercise sessions completed during months four through six was associated with longer sleep duration, less dysfunction during the daytime due to lack of sleep, and non-significantly with less need for sleep medication (see Table 1).

**Figure 1:**
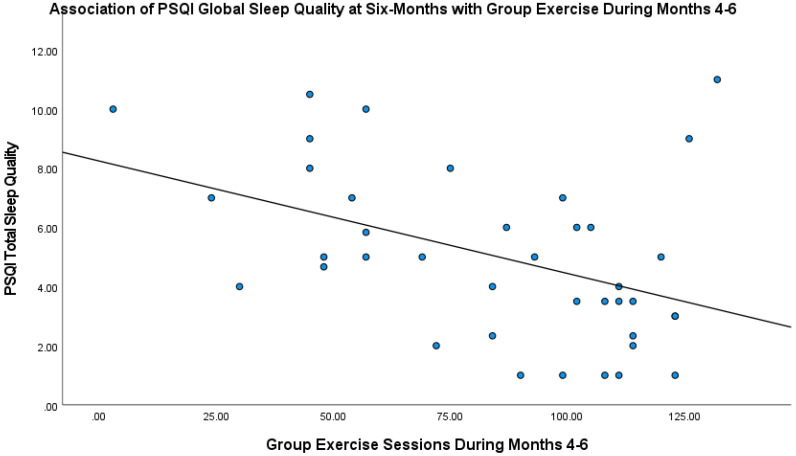
Association of PSQI Global Sleep Quality at Six Months with Group Exercise During Months Four through Six.

At baseline, the mean PSQI Global Sleep Quality score was 6.0 (SD = 2.9, range: 0 to 14), which was slightly over the cutoff to be considered poor sleep quality. At the six-month point, the mean score for all participants had decreased to 5.0 (SD = 2.9, range: 1 to 11). The individual contributions of both group and individual exercise in simultaneously entered regression models predicting pre–post change (baseline to six months) in each PSQI domain and total score are presented in Table 2. Whereas group exercise predicted improvement in total sleep quality, t(33) = −2.84, *p* = 0.008 (see Figure 1), individual exercise was not significantly predictive of total sleep quality, t(33) = −1.33, *p* = 0.19. Group exercise also predicted the individual domain of sleep quality, t(32) = −2.44, *p* = 0.02. Contrary to our hypotheses, the other PSQI subscale scores were not significantly predicted by either group or individual homework exercise frequency during months four through six.

## 4. Discussion

The context of a larger, randomized controlled trial of the effect of adding aerobic exercise to cognitive training among individuals with a recent onset of schizophrenia allowed an investigation of the effects of exercise on sleep quality. Our findings indicate that higher attendance in moderate-intensity group exercise sessions was associated with better sleep quality and sleep duration and fewer reported daytime problems from poor sleep. Further, exercise group attendance was associated with an improvement in sleep quality from baseline to the six-month sleep assessment. Improved sleep quality appears to be yet another benefit of regular exercise among individuals with serious mental illness.

In contrast to our hypothesis, the amount of at-home exercise did not separately predict sleep quality. While the frequency of group and at-home exercise was similar, there were other differences in the two exercise formats. The at-home exercise requirement was for 30 min in length compared with 45 min of group exercise. The group exercise involved moderate- and moderate-to-high-intensity interval training under the direction and encouragement of an enthusiastic certified fitness instructor. Whereas it was encouraged for participants to complete at least moderate-intensity exercise at home, the type of at-home exercise was variable and ranged from lower intensity exercise such as brisk walking to higher intensity exercise activities such as playing sports, running, or following along with an aerobic exercise video. Further, self-report of at-home exercise was likely not as accurate as the in-group exercise attendance, which was verified by the group instructor. This would lead to larger measurement error for the frequency of at-home completion. So, in addition to a concern that patients may have over-reported the frequency of at-home exercise, there is also the factor that the at-home exercise was not as long and more variable in intensity than the instructor-led group exercise. Therefore, the full benefit of exercising might not have been obtained for individual exercise sessions. All of these limitations might have led to the lack of significant findings for analyses involving at-home sessions.

It is difficult to untangle the dual effects of both cognitive training and exercise on sleep in this study because both interventions were provided on the same clinic day, so patients tended to participate in both, not one or the other. It is possible that greater participation in the multiple aspects of the treatment program altogether, with the exception of individual exercise, was associated with better sleep quality. It is also possible that patients who slept better were more likely to engage in exercise as well as other aspects of treatment than those with poor sleep quality. The pattern of associations in this study suggest that exercise improves sleep quality, but the direction of causality cannot be determined.

Poor sleep quality in non-psychiatric populations has been linked to daytime fatigue, cognitive fogginess, lowered alertness, and car accidents, and other adverse outcomes [12,13]. A meta-analysis based on multiple studies concluded that physical exercise can improve sleep among individuals without serious mental illness [14]. A meta-analysis of eight studies of exercise interventions amongst individuals with mental disorders showed that exercise has a beneficial effect on sleep quality [15]. However, none of the studies involved patients with schizophrenia. We have demonstrated that a group of young adults with schizophrenia can be engaged in a regular exercise program, even during the tumultuous early course of the disorder. Further, we have shown that the amount of exercise was associated with improvement in sleep quality. Despite a growing body of evidence suggesting that participation in regular exercise programs shows promising effects on physical health, cognition, and negative symptoms for schizophrenia patients [16,17,18], exercise is a relatively unused adjunctive treatment.

Poor sleep quality among individuals with schizophrenia might contribute to the burden of their disorder in a number of ways, including worsening of cognition and functioning during waking hours, and might contribute to symptoms of metabolic syndrome [5,19]. Individuals with psychosis have abnormally high rates of concurrent physical ailments such as cardiovascular disease and symptoms of the metabolic syndrome, which lead to an increased risk of early mortality [20,21]. Exercise, a feasible non-pharmacologic intervention, might improve both sleep quality and these other functional and health concerns that are often concomitant with schizophrenia. Future work will be needed to determine whether the beneficial effects of improved sleep are separable from the effects of exercise itself.

## Figures and Tables

**Table 1:** Correlational Associations Between Sleep (assessed at Six Months) and Number of Group and At-Home Aerobic Sessions Completed.

	Number of Group Exercise Sessions Completed: 4–6 Months (N = 39): Pearson Correlations	Number of At-Home Exercise Sessions Completed: 4–6 Months (N = 39): Pearson Correlations
Sleep Quality	−0.26	0.01
Sleep Latency	0.06	0.16
Sleep Duration	−0.37 *	−0.19
Sleep Efficiency ^1^	----	----
Sleep Disturbance	−0.11	0.07
Need for Sleep Meds	−0.29 †	−0.15
Daytime Dysfunction	−0.52 ***	−0.12
Overall Sleep Quality	−0.44 **	−0.03

Note: Lower levels of PSQI variables represent better sleep. Higher levels of exercise represent more exercise sessions completed. ^1^ Too few non-zero ratings to analyze. † = <0.10, * = *p* < 0.05, ** = *p* < 0.01, *** = *p* < 0.001.

**Table 2:** Final Model of the Prediction of Change in PSQI Sleep Quality from Baseline to Six Months by Exercise using Linear Multiple Regression.

PSQI Scales	F(df)/t(df)	Standardized Coefficients Beta for Individual Variable Contributions	*p*
Sleep Quality			
Overall Model	F(4,29) = 1.5		0.22
Baseline Group Exercise	t(32) = 0.85	0.21	0.40
Baseline Ind. Exercise	t(32) = −0.84	−0.29	0.41
Group Exercise	**t(32) = −2.44**	**−0.55**	**0.02**
Ind. Exercise	t(32) = 1.42	0.48	0.17
Sleep Latency			
Overall Model	F(4,29) = 1.24		0.31
Baseline Group Exercise	t(32) = 0.17	0.30	0.25
Baseline Ind. Exercise	t(32) = 0.05	0.02	0.96
Group Exercise	t(32) = 0.11	0.03	0.91
Ind. Exercise	t(32) = 0.57	0.20	0.58
Sleep Duration			
Overall Model	**F(4,29) = 4.95**		**0.004**
Baseline Group Exercise	**t(32) = 3.33**	**0.72**	**0.002**
Baseline Ind. Exercise	t(32) = −1.31	−0.37	0.20
Group Exercise	t(32) = −0.49	−0.09	0.63
Ind. Exercise	t(32) = −0.20	−0.05	0.85
Sleep Efficiency		Too few changes in ratings to analyze	
Sleep Disturbance			
Overall Model	F(4,19) = 1.15		0.37
Baseline Group Exercise	t(22) = 0.11	0.03	0.91
Baseline Ind. Exercise	t(22) = 0.60	0.22	0.56
Group Exercise	t(22) = 1.68	0.47	0.11
Individual Exercise	t(22) = −1.74	−0.72	0.10
Need for Sleep Meds			
Overall Model	F(4,29) = 2.56		0.06
Baseline Group Exercise	**t(32) = −2.19**	**−0.52**	**0.04**
Baseline Ind. Exercise	t(32) = 0.95	0.31	0.35
Group Exercise	t(32) = −1.32	−0.28	0.20
Individual Exercise	t(32) = 0.48	0.15	0.63
Daytime Dysfunction			
Overall Model	F(4,29) = 1.41		0.26
Baseline Group Exercise	T(32) = −1.57	−0.40	0.13
Baseline Ind. Exercise	T(32) = 1.23	0.43	0.23
Group Exercise	T(32) = −1.08	−0.24	0.29
Individual Exercise	T(32) = −0.70	−0.24	0.49
Overall Sleep Quality			
Overall Model	F(4,30) = 2.32		0.08
Baseline Group Exercise	t(33) = −0.01	0.00	0.99
Baseline Ind. Exercise	t(33) = 0.06	0.02	0.95
Group Exercise	**t(33) = −2.84**	**−0.57**	**0.008**
Individual Exercise	t(33) = −1.33	0.39	0.19

## Data Availability

The de-identified data will be available from the authors upon completion of the study.
